# Maternal western-style diet alters Kupffer cell proportion leading to metabolic dysfunction-associated steatotic liver disease when challenged with western diet in adulthood

**DOI:** 10.3389/fimmu.2025.1698609

**Published:** 2025-12-10

**Authors:** Sarah J. Miller, Rachel C. Janssen, Wanke Zhao, Karen R. Jonscher, Hua Zhong, Christa I. DeVette, Jacob E. Friedman, Kurt A. Zimmerman

**Affiliations:** 1 Department of Internal Medicine, Division of Nephrology, University of Oklahoma Health Sciences Center, Oklahoma City, OK, United States; 2 Harold Hamm Diabetes Center, University of Oklahoma Health Sciences Center, Oklahoma City, OK, United States; 3 Department of Biochemistry and Physiology, University of Oklahoma Health Sciences Center, Oklahoma City, OK, United States; 4 Department of Obstetrics and Gynecology, University of Oklahoma Health Sciences Center, Oklahoma City, OK, United States; 5 Department of Pediatrics, Section of Neonatal-Perinatal Medicine, University of Oklahoma Health Sciences Center, Oklahoma City, OK, United States

**Keywords:** western-style diet, steatotic liver disease, macrophages, MAFLD, developmental programming

## Abstract

Exposure to maternal western-style diet (mWD) is associated with early development of metabolic dysfunction-associated fatty liver disease (MAFLD) in offspring. Kupffer cells (KCs), the main resident macrophage population in the liver, are known to promote MAFLD progression; however, the effects of mWD on KC subtypes, ontogeny, and gene expression in offspring are not fully understood. In this manuscript, we used two different models of mWD exposure and challenge in adulthood to understand the impact of mWD on KC proportion, ontogeny, and gene expression in adult offspring. Our data indicate that in the absence of challenge in adulthood, mWD results in increased KC proportion in offspring, with limited changes in KC ontogeny or liver phenotype. In contrast, mWD mice challenged with WD in adulthood had increased expression of inflammatory (*Nlrp3*) and fibrosis (*Tgfb1*)-related genes compared with chow (CH)-WD-fed mice. Although KC proportion and ontogeny were similar when comparing CH-WD- and WD-WD-fed mice, we found that WD-WD mice had a greater reduction in KC proportion and TdT labeling than CH-WD-fed mice when normalized to their respective maternal diet controls. Similar results were found when mice were weaned onto normal chow and rechallenged with WD later on in adulthood. Bulk RNA sequencing data indicate that KCs from mWD mice rechallenged with WD in adulthood had increased expression of inflammatory and antigen-presenting genes compared with KCs isolated from WD-fed mice lacking mWD exposure. These findings highlight the intergenerational repercussions of mWD on liver phenotype as well as KC proportion and ontogeny and provide novel insight into the mechanisms dictating MAFLD.

## Highlights

Exposure to a high-fat maternal WD (mWD) in the perinatal period predisposes offspring to develop MAFLD in adulthood; however, the impact of mWD exposure on Kupffer cell number, ontogeny, and gene expression in offspring needs further clarification. In this manuscript, we report that perinatal exposure to mWD results in altered KC proportions with modest changes in KC ontogeny whereas mWD with WD in adulthood leads to increased expression of inflammatory and fibrosis-related genes. Thus, perinatal exposure to mWD results in long-lasting alterations in KC development and response to WD challenge in offspring.

## Introduction

Maternal factors including maternal overnutrition can have long-lasting effects on offspring health and disease risk later in life, leading to changes in organ structure, function, and metabolism, and is referred to as the Developmental Origins of Health and Disease (DOHaD) (1–5). The impact of maternal high-fat diet exposure and maternal obesity on the development of metabolic disease in offspring has been attributed, in part, to its impact on immune cell phenotype and function during development (6–8). A maternal western-style diet-(mWD) high in fat and sugar and/or maternal obesity in mice was found to restrict expansion of fetal hematopoietic stem cells, a phenotype that persisted well into adulthood in offspring (9, 10). In non-human primates, mWD resulted in a pro-inflammatory polarization of bone-marrow-derived macrophages in offspring, a phenotype which persisted for 2.5 years postnatally (8). Additionally, maternal obesity or poor diet is implicated in driving an altered metabolic programming in liver Kupffer cells (KCs), which underlies development of metabolic-dysfunction-associated steatotic liver disease (MAFLD) in young offspring, even in the absence of obesity (10, 11). As fate mapping studies have shown that KCs in healthy adult animals are over 90% embryonically derived, with <10% of cells originating from adult bone marrow monocytes (12, 13), these cells are primed to act as intergenerational messengers when exposed to environmental challenges *in utero* such as mWD (14). Importantly, the proportion of embryonic versus bone marrow derived KCs can change with inflammation or liver injury, as circulating monocytes infiltrate and engraft into the existing KC niche (15, 16).

In the homeostatic murine liver, two embryonically derived subtypes of KCs have been proposed: KC1s, which express genes associated with immune-related pathways, and KC2s, which express genes associated with lipid metabolism (17, 18). While the exact mechanism driving formation of these subtypes is unknown, KCs as a whole exhibit significant heterogeneity and plasticity, resulting in dynamic changes in the number, phenotype, and function of these cells during disease, even in the absence of mWD exposure (18, 19). Many MAFLD mouse models show altered KC numbers and ontogeny, with an increased bone marrow monocyte contribution to the KC niche as well as changes to the proportion of KC1s versus KC2s (20–23). Interestingly, the function of KCs in disease can differ depending on their origin. For example, lipid-associated macrophages (LAMs) are a bone marrow-derived population found in steatotic livers (23). While the embryonically derived resident KCs can take on a LAM-like phenotype and exhibit functional redundancy with monocyte-derived LAMs, LAM-like KCs have an increased capacity for phagocytosis as well as altered gene expression compared with monocyte-derived LAMs (23).

Given the reported effects of ontogeny on KC function, as well as the known impact of maternal obesity on macrophage phenotypes and liver health, we sought to investigate whether maternal WD exposure impacts the function and ontogeny of KCs in offspring using the recently developed GMP-specific fate mapping mouse model (*Ms4a3^Cre/Cre^ Rosa stop^f/f^ TdT*), which labels Ly6^hi^ monocytes and Ly6c^hi^ monocyte-derived macrophages in the liver (13). Here, we report that mWD exposure during gestation and lactation results in an increased KC proportion in chow (CH)-fed offspring with marginal effects on KC origin and liver phenotype. We also found that maternal WD exposure coupled with WD feeding in offspring led to a reduced proportion of total KCs and KC1s, and these reductions correlated with increased inflammatory and fibrotic gene expression. Lastly, bulk RNA sequencing analysis revealed that mWD exposure coupled with postnatal WD feeding in offspring leads to increased expression of genes associated with inflammation and antigen presentation in KCs compared with WD-fed mice lacking mWD exposure. These findings highlight the long-lasting impacts of maternal health and diet and provide direction for future studies investigating the mechanisms driving MAFLD pathogenesis.

## Methods

### Animal studies

*Ms4a3^Cre^* mice were the kind gift of Dr. Florent Ginhoux. *Rosa stop^fl/fl^ TdT* mice (B6.Cg-*Gt(ROSA)26Sor^tm14(CAG-TdTomato)Hze^*/J; Stock No: 007914) were purchased from The Jackson Laboratory (Bar Harbor, Maine). Mice were bred and maintained in-house, under pathogen-free conditions, in a 12:12 light:dark cycle. All studies were conducted in full accordance with AAALAS, NIH, and USDA guidelines, with approval of the University of Oklahoma Health Institutional Animal Care and Use Committee. Studies were conducted under IACUC protocol 20-022-SACHX.

### Maternal HFD studies

Adult female *Ms4a3^Cre/Cre^* or *Rosa stop^fl/fl^ TdT* mice were randomly assigned to a high-fat “Western style” diet (WD) obtained from Envigo Teklad Diets (TD.88137: 15.2% protein, 42.7% carbohydrates, and 42% fat; 4.5 kcal/g) for 2 weeks before being used in breeding. Age-matched littermates were used as a control and were maintained on standard chow diet (CH) purchased from LabDiet (PicoLab Rodent diet 20 5053: 24.5% protein, 62.4% carbohydrates, and 13.1% fat; 3.02 kcal/g) (**Supplementary Figures S1A, B**). Dams were maintained on selected diet (mCH or mWD) throughout pregnancy and lactation. Offspring from the continual challenge experiments were weaned onto either CH or WD, aged to approximately 12 weeks, and euthanized for analysis. The offspring used in rechallenge experiments were all weaned onto standard chow; at 12 weeks of age, mice were randomly split into two groups, with one group receiving standard chow and the other receiving WD. These mice were maintained on this diet until euthanasia at approximately 16 weeks of age. At all harvest timepoints, mice were euthanized under non-fasted conditions with carbon dioxide at a 70% flow rate (4.7 L/min); chamber flow was increased incrementally through gradual filling for all animals. Following euthanasia, blood and liver were harvested for downstream processing or stored at −80 °C. In preliminary experiments, steatosis was not observed in female mice in any experimental conditions; thus, only male mice were used for the studies in this manuscript (24).

### Plasma analyses

Blood was collected from the portal vein in a syringe rinsed with 0.5 M EDTA, incubated on ice for 30 min, and centrifuged at 1,800 x g for 10 min at 4 °C. The upper plasma layer was collected and stored at −80 °C. Plasma glucose was measured by Glucose (GO) Assay kit (Sigma-Aldrich, Catalog#: GAGO20) following the manufacturer’s instructions using scaled-down sample and reagent amounts for a 96-well microplate assay. Plasma insulin was analyzed using a mouse insulin ultrasensitive ELISA kit (Crystal Chem, Catalog#: 90080) following the manufacturer’s instructions.

### Flow cytometry

Flow cytometry was performed on liver tissue using established protocols (25). In brief, following euthanasia and blood collection, mice were perfused with PBS and the median lobe of the liver was removed. Tissue was then minced followed by digestion in 1 mL digestion buffer comprised of 5 mg/mL collagenase Type IV (Sigma-Aldrich, Catalog#: NC0217889) and 100 U/mL DNase I (Sigma-Aldrich, Catalog#: D5025-15KU) for 30 min at 37 °C. Cells were then passed through a 70-μm strainer, and red blood cells were lysed using ACK lysis buffer. The remaining cells were resuspended in 1 mL PBS containing 1% BSA with Fc receptor blocking solution for 30 min on ice, before 2 × 10^6^ cells were stained using the primary antibodies indicated in **Supplementary Table S1** for 30 min at room temperature. After a final wash, cells were fixed in 2% paraformaldehyde for 30 min on ice before a final resuspension in D-PBS. Samples were run on a Cytek Aurora and analyzed using FlowJo version 10.10.0 software. The gating strategy used to identify total KCs, KC1s, KC2s, and TdT+ cells is shown in **Supplementary Figure S3**.

### Bulk RNA sequencing

Cells were prepared as described in the flow cytometry section. After antibody staining. (**Supplementary Table S1**) for 30 min, cells were spun, washed with 1% BSA, and sorted using a Becton–Dickenson FACSAria II. Approximately 50,000 live TdT^+^ KCs and TdT^−^ KCs were sorted from individual mice (n=2 mCH-CH-WD, n=5 mWD-CH-WD) into individual tubes containing TRIzol.

### Bulk RNA processing

RNA was isolated using a standard TRIzol extraction protocol, and libraries were generated using QuantSeq protocol on an Ilumina NovaSeq 6000 system (26). Generated Fastq files were subjected to a data cleaning step involving trimming to remove low-quality bases, eliminating short/low-quality reads and polyA/G tails, and trimming adapters using the FastP tool (27). We used multiQC as a means to confirm alignment with GENCODE reference genome GRCm39/M34 using the STAR software package (28). Following alignment, differential expression was then applied to the count files using DESeq2 (29).

### qPCR

RNA was isolated from frozen liver (~25 mg) using Direct-zol RNA Miniprep Kit (Zymo Research) per instructions. cDNA synthesis using iScript RT Supermix (Bio-Rad, catalog# 1708840) and quantitative PCR using the PowerUp SYBR mix (Thermo Fisher, Catalog#: A25743) were performed on a QuantStudio 6 Real-Time PCR System (Thermo Fisher) following the manufacturer’s instructions, and data were normalized to *Rn18s* using the comparative Ct method. Primers used are detailed in **Supplementary Table S2**.

### Triglyceride analysis

Triglycerides were measured in livers using Infinity Triglyceride Reagent (Thermo Fisher, Catalog#: TR22421) following the manufacturer’s instructions.

### H&E, picrosirius, and oil red O staining

Liver tissue was fixed in 4% paraformaldehyde for 24 h and then samples were transferred to 70% ethanol, paraffin-embedded, and cut in 5-μm sections onto glass slides. Liver sections were de-paraffinized, rehydrated, and stained with hematoxylin and eosin (H&E) or picrosirius red by the OUHSC Stephenson Cancer Tissue Pathology Core. For oil red O staining, fresh liver tissue was embedded in optimal-cutting-temperature compound and cryo-sectioned at 5 μm, fixed with formalin for 5 min, and then stained in freshly prepared 0.5% oil red O in propylene glycerol. Sections were washed with PBS, counterstained with hematoxylin, and mounted using VectaMount AQ aqueous mounting medium (Vector Labs). Slides were visualized using a Cytation 5 microscope and Gen5 imaging software (Agilent).

### Quantification of hepatocellular ballooning

H&E-stained liver sections were analyzed for hepatocellular ballooning by a technician blinded to the treatment modality. Images were analyzed and assigned a score of 0 (no ballooning present), 1 (mild to few ballooning cells evident), or 2 (marked or many ballooning cells evident), as defined by field standards (30–32). Scoring for individual mice was done using at least two separate images for each animal, and the average image score was used.

### Quantification of picrosirius staining

Picrosirius-stained liver sections were analyzed for total picrosirius positive area (PSR+). Using ImageJ (33), picrosirius-positive areas were identified by thresholding grayscale images. The total PSR+ area was quantified as the PSR+ area per image divided by the total area of the liver in the image field. This quantification was done using at least two separate images for each animal. Quantification and analysis were performed by a technician blinded to the treatment modality.

### Statistical analysis

All samples are shown as mean ± SEM. Statistical differences were determined by two-way ANOVA followed by Fishers Least Significant Difference (LSD) test. Fishers LSD test was selected based on both the increased power it provides and our sample size. ANOVA P values for the interaction, maternal diet (mDiet), and offspring diet (oDiet) are provided on each respective graph. All tests were conducted using GraphPad Prism version 10.6.0.

## Results

### Exposure to mWD enhances inflammation and fibrosis in the liver when offspring are weaned onto a WD

**Figure 1:**
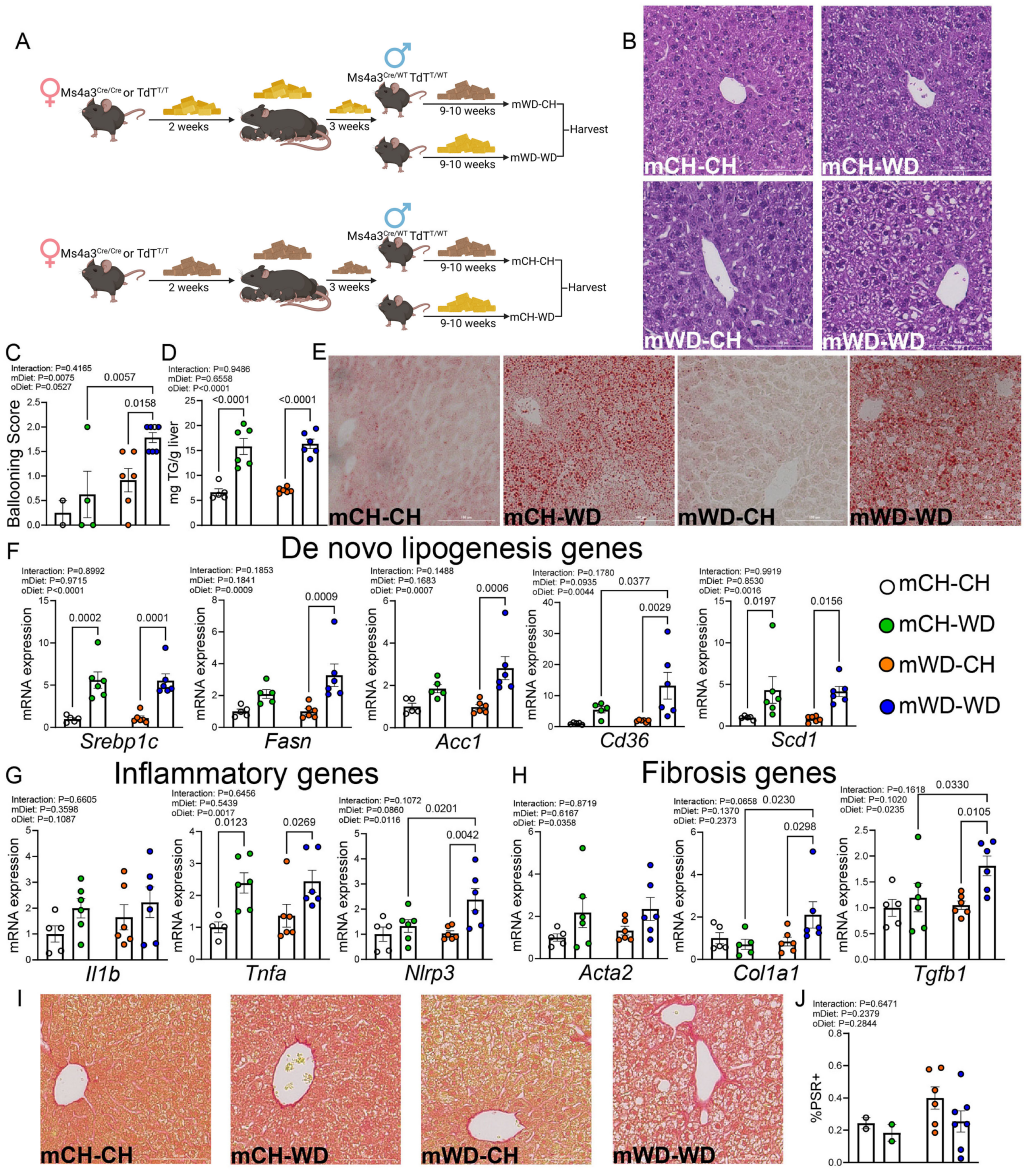
The impact of maternal WD and continuous postnatal WD exposure on liver phenotype. **(A)** Experimental schematic depicting study design. Groups are named as (diet of dam)-(diet of offspring post weaning), i.e., mCH-CH represents a chow (CH) fed dam-CH fed offspring. Groups are mCH-CH, mCH-WD, mWD-CH, and mWD-WD. **(B)** Representative H&E-stained liver sections from all experimental groups. **(C)** Average hepatocellular ballooning scores across groups, where a score of 0=none present, 1= mild/few cells affected, and 2=marked/many cells affected. **(D)** Liver triglyceride (TG) levels across experimental groups. **(E)** Representative images of oil red O-stained liver sections from all groups. mRNA expression of *de novo* lipogenesis genes **(F)**, inflammatory genes **(G)**, and fibrosis genes **(H)**. Gene expression was normalized to *Rn18s* expression, with fold-change calculations based on the mCH-CH group. **(I)** Representative picrosirius red-stained liver sections from all experimental groups. **(J)** Quantification of the percentage picrosirius positive (PSR+) area out of the total image area, calculated from picrosirius-stained images as seen in **(I)** Data are shown as the means ± S.E.M., two-way ANOVA with Fisher’s LSD test **(C, D, F-H, J)**. n=6–7 per group, male mice; each dot represents one animal **(C, E-G)**. In all images **(B, E, I)**, scale bar represents 100 μm. For **(C, J)**, analysis was done using n=2 fields of view per animal, n=2–7 mice per group, male mice. Each dot represents the average of the individual animal.

To test the impact of maternal *vs*. post-weaning WD exposure, or their combination, on liver phenotype or KC ontogeny in young adult offspring, we crossed mice expressing cre recombinase downstream of the endogenous *Ms4a3* promoter (*Ms4a3*^cre/cre^) to mice containing a stop flox TdT reporter (Stop^f/f^ TdT) inserted in the Rosa locus to generate *Ms4a3*^cre/wt^ Stop^f/f^ TdT reporter mice. Importantly, the *Ms4a3* promoter is exclusively activated in granulocyte-monocyte progenitors (GMPs) including Ly6c^hi^ monocytes and their progeny, allowing for quantification of monocyte input into the KC niche (13). Eight-week-old female *Ms4a3*^cre/cre^ or Rosa stop^f/f^ TdT mice were fed a high-fat, high-sugar WD (**Supplementary Figures S1A, B**) for 2 weeks prior to mating to CH-fed male *Ms4a3*^cre/cre^ or *Rosa stop^f/f^ TdT* mice (**Figure 1A**). All offspring were exposed to maternal diets through lactation and weaned at 3 weeks of age onto either CH or WD until harvest at 12 weeks of age (**Figure 1A**). At the time of harvest, under non-fasted conditions, mWD did not significantly impact body weight, plasma insulin, or glucose levels, although mice fed a postnatal WD had elevated body weight compared with CH-fed littermate controls, independent of maternal diet (**Supplementary Figures S2A-C**).

Histological examination of H&E-stained sections showed significant hepatocellular ballooning in mWD-WD mice compared with mCH-WD or mWD-CH mice (**Figures 1B, C**). Analysis of liver triglycerides (TGs) showed enhanced lipid accumulation in mCH-WD and mWD-WD groups, which was confirmed by oil red O staining (**Figures 1D, E**), indicating that diet in adulthood had the greatest effect on TG accumulation. Expression of multiple *de novo* lipogenesis genes were increased in mWD-WD offspring (*Srebp1c, Fasn, Acc1, Cd36, Scd1*), and to a lesser degree in mCH-WD offspring, compared with mCH-CH and mWD-CH controls (**Figure 1F**). We also found increased expression of the inflammasome-associated gene *Nlrp3* in mWD-WD mice compared with mCH-WD and mWD-CH mice as well as increased expression of *Tnfa* in both mCH-WD and mWD-WD mice compared with their respective controls (**Figure 1G**). Analysis of fibrotic genes showed that mWD-WD mice showed significantly increased expression of *Tgfb1* and *Col1a1* compared with mCH-WD and mWD-WD controls (**Figure 1H**), although analysis of collagen protein via picrosirius red staining showed no significant differences between groups (**Figures 1I, J**). Collectively, these data indicate that WD in adulthood results in increased lipid accumulation and expression of lipogenesis genes. Continual WD exposure of mWD offspring results in increased inflammatory (*Nlrp3*) and fibrosis (*Tgfb1*, *Col1a1*) related genes compared with mice exposed to WD in adulthood only.

### Exposure to mWD alters Kupffer cell proportions in offspring weaned on WD

**Figure 2:**
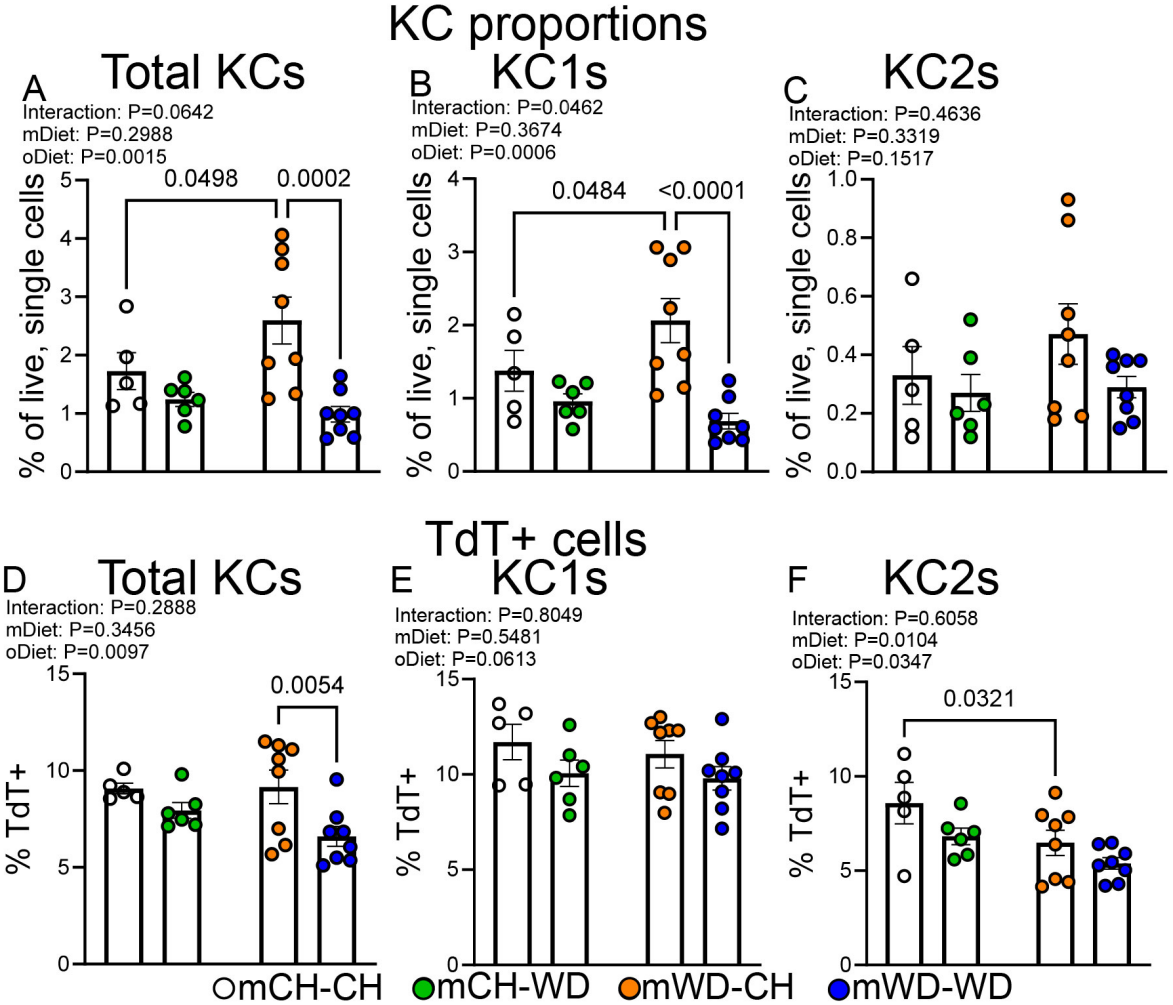
The impact of maternal WD and continuous postnatal WD exposure on Kupffer cell proportions and ontogeny. Quantification of total Kupffer cells (KCs) **(A)**, CD206^−^ KCs (KC1s) **(B)**, and CD206^+^ KCs (KC2s) **(C)**, shown as a percentage of all live, single cells. Quantification of TdTomato-positive (TdT^+^) total KCs **(D)**, KC1s **(E)**, and KC2s **(F)**, shown as a percentage of the parent population. Data are shown as the means ± S.E.M., two-way ANOVA with Fishers LSD test. n=5–8 per group, male mice; each dot represents one animal.

Approximately ~95% of bone-marrow derived Ly6c^hi^ monocytes are TdT+ in *Ms4a3*^cre/wt^ Stop^f/f^ TdT reporter mice (13), allowing for quantification of monocyte input into the KC niche in healthy and diseased mice. In contrast, embryonically derived KCs remain unlabeled. To this end, we analyzed KC proportions and TdT labeling via flow cytometry using the gating strategy found in **Supplementary Figure S3**. We further subset KCs into KC1s and KC2s based on the expression of CD206, as previously reported (17). Despite the lack of an observable phenotype, we found that mWD-CH mice showed increased proportions of total KCs and KC1s compared with mCH-CH fed mice (**Figures 2A-C**). We also found that total KC and KC1 ontogeny was not impacted by mWD in the absence of continual challenge (**Figures 2D-F**). In contrast, we found that mice born to mWD dams that were continually challenged with WD in adulthood had greater reduction in KC proportion and TdT labeling compared with mCH-WD fed mice in relation to respective maternal diet controls (**Figures 2A-F**). We did not find any significant difference in KC proportion or TdT labeling in any cell type when comparing mCH-WD with mWD-WD fed mice (**Figures 2A-F**). In sum, these data indicate that mWD in the absence of postnatal WD (mWD-CH) alters KC proportions despite the lack of obvious phenotypic changes in the liver. Furthermore, we found mWD mice continually challenged with WD in adulthood had a greater reduction in KC proportion and TdT labeling than mCH-WD mice when compared with respective maternal diet controls.

### Perinatal exposure to mWD accelerates liver disease after adult rechallenge with a WD

**Figure 3:**
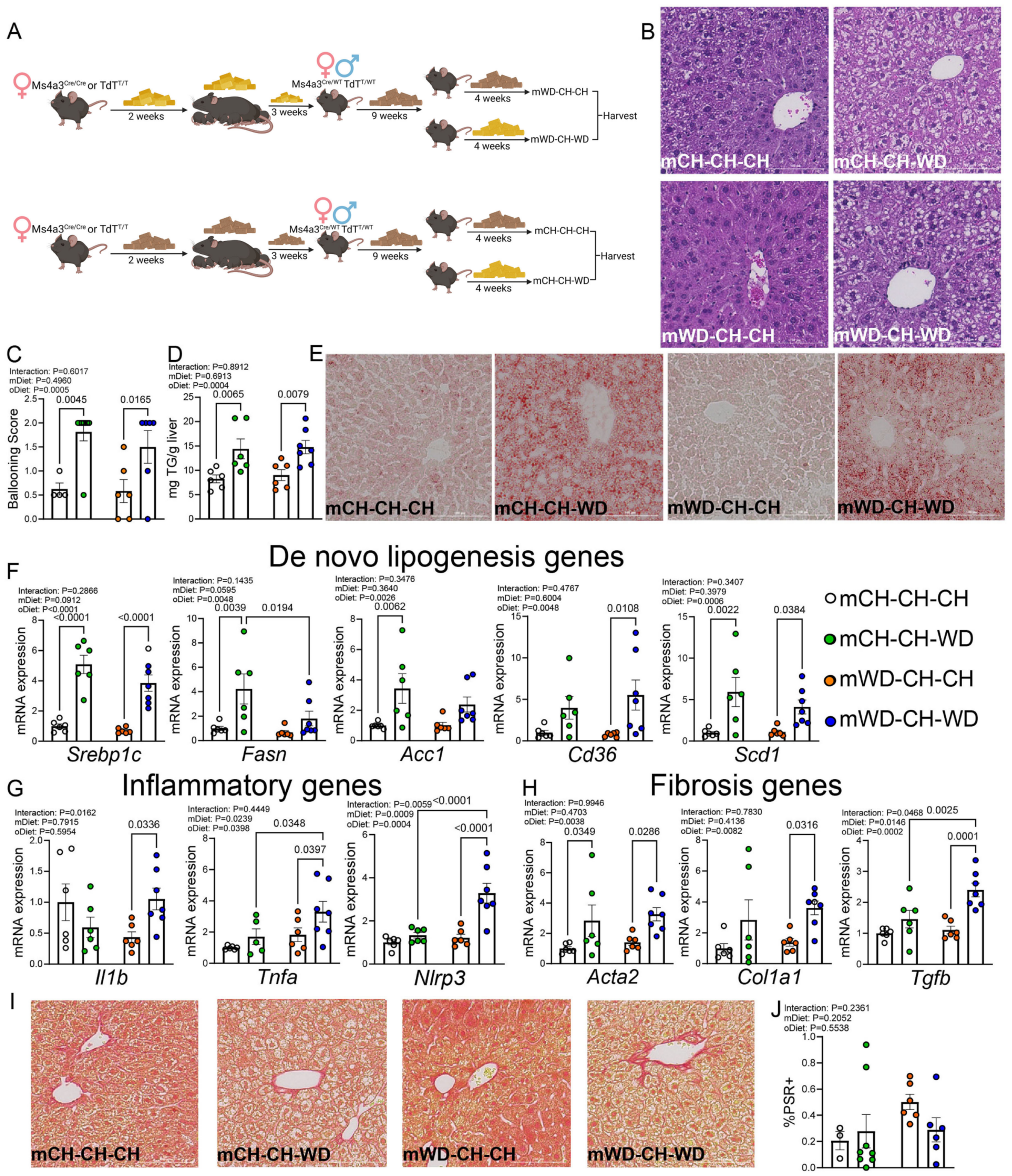
The impact of mWD with delayed WD rechallenge on liver phenotype. **(A)** Experimental schematic detailing study design. Groups are named as (diet of dam)-(diet of offspring 3–12 weeks)-(diet of offspring 12–16 weeks), i.e., mCH-CH-WD represents a CH fed dam-CH fed offspring-WD rechallenged offspring. Groups are mCH-CH-CH, mCH-CH-WD, mWD-CH-CH, and mWD-CH-WD. **(B)** Representative images of H&E-stained liver sections from all groups. **(C)** Average hepatocellular ballooning scores from H&E-stained images as seen in **(B, D)**. Liver triglyceride levels at time of harvest. **(E)** Representative oil red O-stained liver section images from all groups. mRNA expression of *de novo* lipogenesis genes **(F)**, inflammatory genes **(G)**, and fibrosis genes **(H)**. Gene expression was normalized to *Rn18s* expression, with fold-change calculations based on the mCH-CH-CH group. **(I)** Representative images of picrosirius red-stained liver sections from all groups. **(J)** Quantification of picrosirius positive (PSR+) area out of total image area, calculated from picrosirius-stained images such as those seen in **(I)** Data are shown as the means ± S.E.M., two-way ANOVA with Fisher’s LSD test **(C, D, F-H, J)**. n=6–7 per group, male mice, with each dot representing one animal. In all images shown **(B, D, I)**, scale bar represents 100 μm. For **(C, J)**, quantification was done using n=2 fields of view per animal, n=3–8 mice per group, male mice. Each dot represents the average for an individual animal.

Previous data indicate that mWD offspring weaned onto a CH diet have enhanced MAFLD phenotypes when rechallenged with WD later in life (11). To test the impact of rechallenge with WD on KC proportions and ontogeny, pups born to WD- and CH-fed female mice were weaned onto CH diet for 9 weeks followed by rechallenge with WD or CH for an additional 4 weeks (from 12–16 weeks of age) (**Figure 3A**). Offspring are depicted as mCH-CH-CH, mCH-CH-WD, mWD-CH-CH, or mWD-CH-WD, indicating what diet the dam was fed, what diet the offspring were weaned onto, and what diet the offspring were fed from 12–16 weeks of age. Body weight in offspring at time of harvest was increased in mCH-CH-WD and mWD-CH-WD mice compared with mCH-CH-CH and mWD-CH-CH mice, again indicating that postnatal WD challenge drove weight gain (**Supplementary Figure S4A**). Non-fasted serum insulin at time of harvest was increased in mCH-CH-WD mice compared with all other groups (**Supplementary Figure S4B**), whereas serum glucose levels were increased in mWD-CH-WD mice (**Supplementary Figure S4C**), potentially indicating that mWD with WD challenge in adulthood predisposed these mice to the insulin resistance that is often seen in juvenile obesity (34, 35). H&E-stained sections showed normal hepatocyte morphology in the mCH-CH-CH and mWD-CH-CH groups, whereas hepatocyte ballooning was observed in both mCH-CH-WD and mWD-CH-WD mice (**Figures 3B, C**). Analysis of liver triglycerides (TGs) showed enhanced lipid accumulation in the mCH-CH-WD and mWD-CH-WD groups compared with offspring fed CH diet in adulthood, which was confirmed by oil red O staining (**Figures 3D, E**). Expression of *de novo* lipogenesis genes was increased in mice exposed to WD in adulthood, independent of maternal diet (**Figure 3F**). Combined, these findings indicate that hepatocyte morphology, liver triglyceride levels, and lipogenesis-related gene expression are primarily impacted by diet in adulthood rather than maternal diet.

We again found that expression of *Nlrp3* and *Tnfa* was increased in mWD-CH-WD mice compared with mCH-CH-WD and mWD-CH-CH groups (**Figure 3G**). Additionally, in mWD-CH-WD mice, increased expression of fibrosis-associated genes *Col1a1* (versus mWD-CH-CH) and *Tgfb1* (versus mCH-CH-WD and mWD-CH-CH) was observed (**Figure 3H**), whereas picrosirius red staining was not different between groups (**Figures 3I, J**). Together, these data indicate that WD challenge in adulthood alone leads to worsened hepatocyte ballooning and liver triglyceride levels, whereas WD rechallenge in adulthood in the context of mWD results in increased expression of inflammatory and fibrosis-related genes compared with mWD-CH-CH- and mCH-CH-WD-fed mice.

### Exposure to mWD followed by rechallenge with WD in adulthood alters Kupffer cell frequency and ontogeny

**Figure 4:**
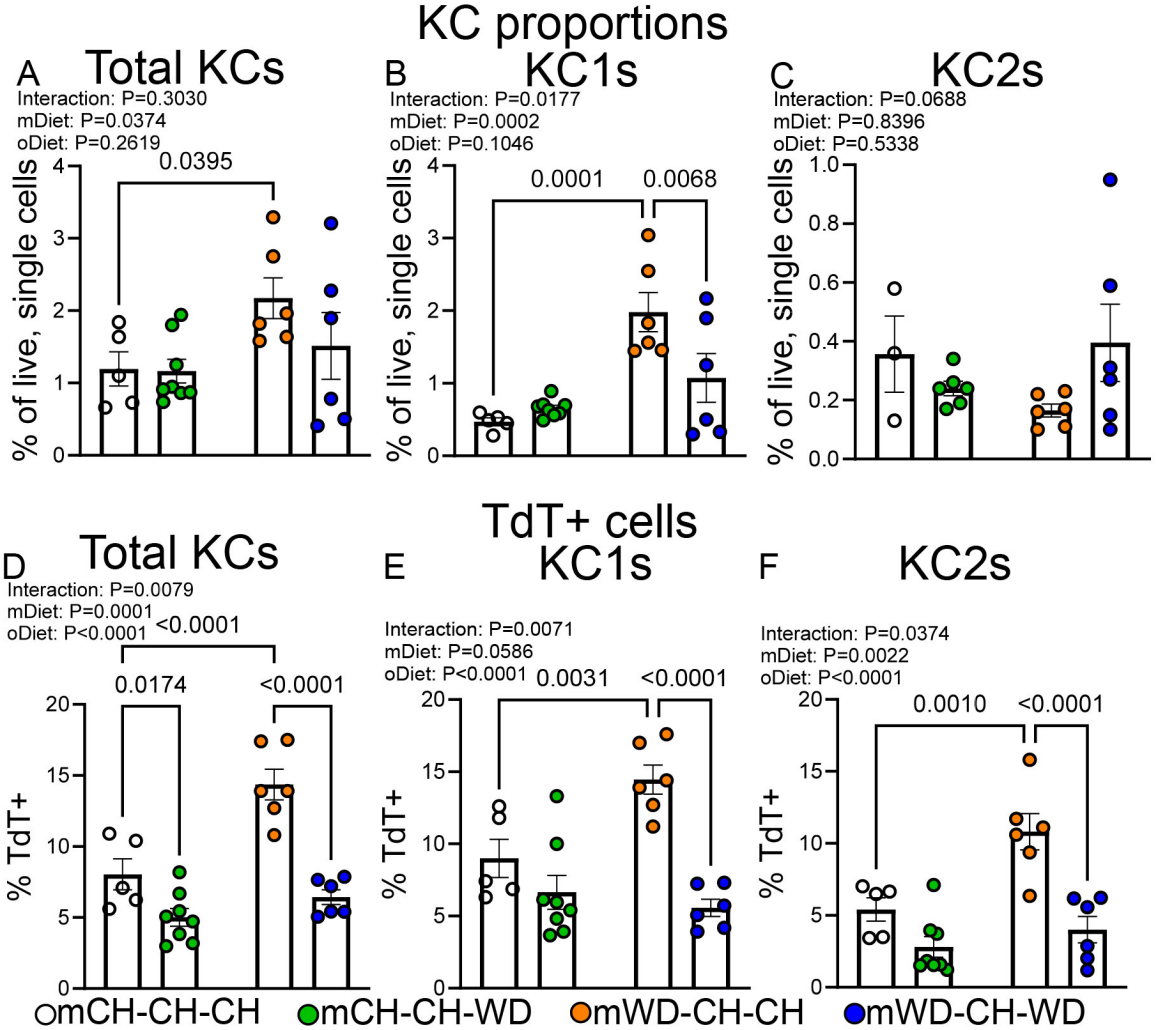
Maternal WD with delayed WD challenge alters Kupffer cell proportions and ontogeny. Quantification of total KCs **(A)**, KC1s **(B)**, and KC2s **(C)**, shown as a percentage of all live, single cells. Quantification of TdT^+^ total KCs **(D)**, KC1s **(E)**, and KC2s **(F)**, shown as a percentage of the parent population. Data are shown as the means ± S.E.M., two-way ANOVA with Fisher’s LSD test. n=5–8 per group, male mice; each dot represents one animal.

To determine the impact of adult WD rechallenge on the proportion and origin of KCs, we again analyzed KCs using flow cytometry. Similar to what was observed in our first experiment, mWD-CH-CH offspring had increased proportions of total KCs and KC1s compared with other groups, despite the lack of an observable liver phenotype (**Figures 4A-C**). We also found that total KCs, KC1s, and KC2s isolated from mWD-CH-CH mice had increased TdT labeling compared with other groups, indicating a monocyte influx into the KC niche in these mice (**Figures 4D-F**). In contrast, we found that mice born to mWD dams that were challenged in adulthood with WD had a greater reduction in KC proportion and TdT labeling than mCH-CH-WD mice when compared with their respective maternal diet controls (**Figures 4A-F**). However, we did not find any significant difference in KC proportion or TdT labeling in any cell type when comparing mCH-CH-WD- to mWD-CH-WD-fed mice (**Figure 4**). These data indicate that mWD-CH-CH offspring have increased proportions of total KCs and KC1s as well as increased TdT labeling despite not having an observable liver phenotype, whereas a rechallenge with WD in adulthood resulted in reduced total KC and KC1 proportion and TdT labeling. These results show that maternal diet alone drives an increase in KC proportions and monocyte input to the KC niche, whereas mWD with WD rechallenge in adulthood abrogates these changes.

### Perinatal exposure to WD alters KC transcriptional signatures after rechallenge with a WD

**Figure 5:**
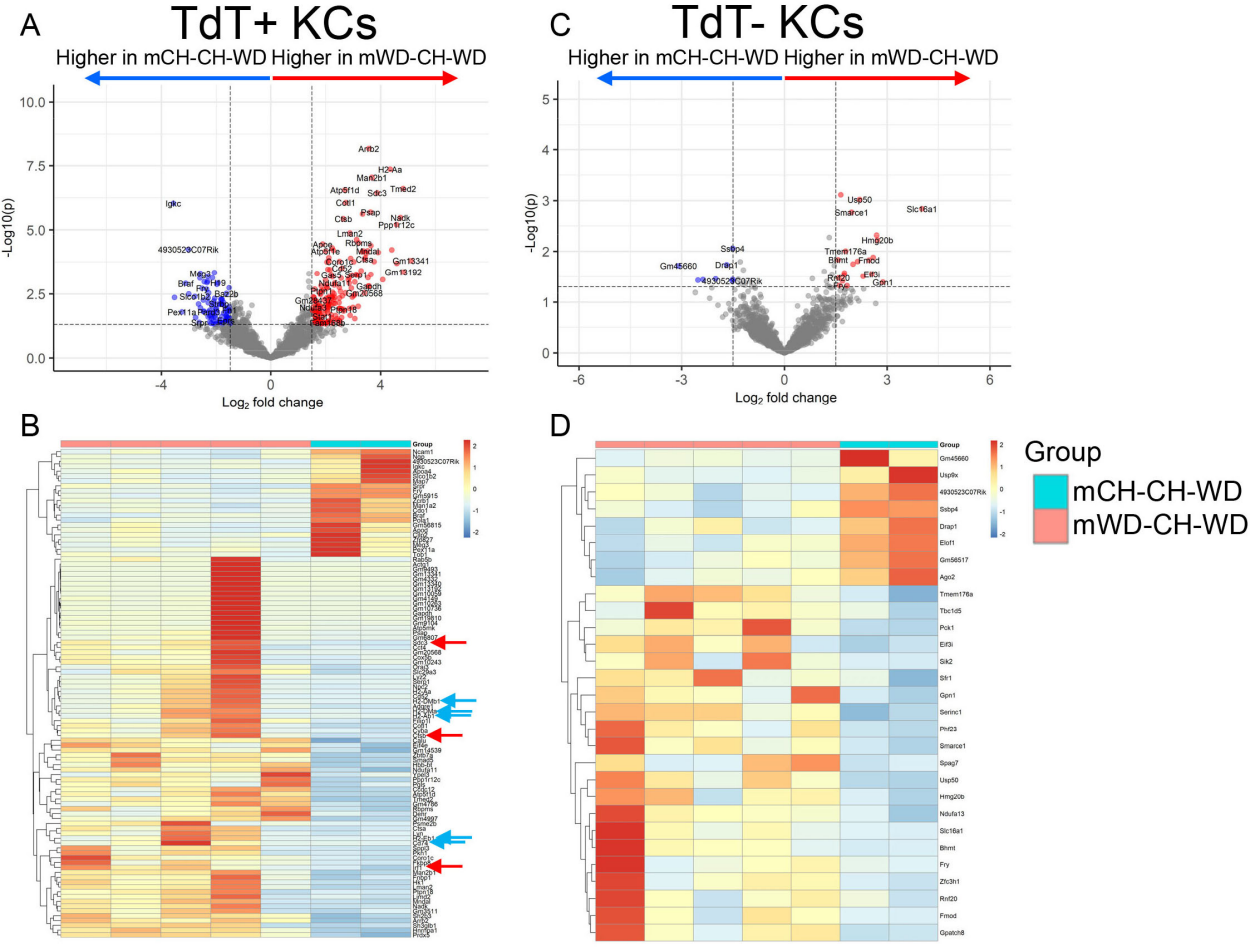
Rechallenge with WD diet increases pro-inflammatory gene expression in offspring from WD-fed dams. Volcano plots showing top differentially expressed genes that were enriched in TdT^+^ KCs **(A)** and TdT^−^ KCs **(C)** when comparing mCH-CH-WD and mWD-CH-WD groups. Heatmap of differentially expressed genes found in mCH-CH-WD and mWD-CH-WD groups from TdT^+^ KCs **(B)** and TdT^−^ KCs **(D)**. n=2–5 per group, male mice.

Disruptions in the immune system due to exposure to mWD has been suggested to drive the early onset of MAFLD in offspring (4), but the disease mechanisms are not fully understood. Our data indicate that continual challenge or rechallenge of offspring born to WD-fed dams results in increased expression of inflammatory genes compared with mice from CH-fed dams who were only fed a WD in adulthood, despite the fact that KC proportions and ontogeny were similar between groups. These data suggest that when pups are exposed to a WD *in utero* and during lactation, they are poised to produce increased pro-inflammatory cytokines upon rechallenge, resulting in a worsened MAFLD phenotype. To test this hypothesis, we performed bulk RNA sequencing on TdT^+^ and TdT^−^ KCs isolated from mCH-CH-WD and mWD-CH-WD mice. In line with phenotype data, we found that TdT^+^ KCs from the mWD-CH-WD group had increased expression of several pro-inflammatory genes including *Irf1, Sdc3*, and *Ctsb* (**Figures 5A, B**, red arrows) (36–38). We also found that TdT^+^ KCs from the mWD-CH-WD group had increased expression of antigen presentation genes including *Cd74, H2-Eb1, H2-Ab*, and *H2-DMb1* (**Figures 5A, B**, blue arrows). In contrast, we found minimal differences in TdT^−^ KCs between groups (**Figures 5C, D**), suggesting that the monocyte-derived TdT^+^ KCs are the population most impacted by mWD.

## Discussion

Our data indicate that mice fed WD in adulthood had increased hepatocyte ballooning, triglyceride accumulation, and expression of lipogenesis genes, independent of maternal diet status. We also found that the proportion of total KCs and KC1s was increased in WD-CH mice, despite the lack of an obvious liver phenotype. mWD offspring challenged with WD in adulthood had increased expression of inflammatory and fibrotic-related genes compared with mice fed WD in adulthood only. These mice also had a greater reduction in KC proportion and TdT labeling when compared with mice fed a WD in adulthood only (in relation to their respective maternal diet controls). Although we found reduced KC proportion and TdT labeling in mWD mice challenged in adulthood, KCs isolated from these mice had enhanced expression of inflammatory and antigen-presenting genes, suggesting that the KCs remaining in the liver are programmed by mWD to have an enhanced inflammatory response following challenge in adulthood. Thus, KCs serve as intergenerational messengers relaying information from *in utero* to adulthood.

The decrease of total KCs and KC1s seen in the mWD-WD and mWD-CH-WD groups in our data aligns with previously reported results from a number of studies where KC numbers were found to be dramatically decreased in diseases including MAFLD, bacterial infection, viral hepatitis, liver cirrhosis, and acute liver injury (19–21, 40–44). As KCs have been proposed to be protective in MAFLD (45), a decrease in this cell population may contribute to worsened disease pathology observed in our studies. Furthermore, KC2s have been implicated in the regulation of lipid processing in the liver via CD36 and targeted depletion of these cells prevented diet-induced obesity in mice, suggesting that KC2s promote MAFLD (17). The fact that we found reduced total KC proportions but stable KC2 proportions in mWD mice rechallenged with WD in adulthood, along with a modestly worsened phenotype, supports that idea that KCs restrict MAFLD whereas KC2s promote MAFLD. While the mechanisms governing KC population reduction in liver diseases are not clearly defined, one mechanism proposed by Guilliams et al. (19) theorizes that the resident KCs may activate and respond to an initial liver insult (mWD in our studies) but cannot maintain this activation state and, thus, die off. Our data showing higher expression of inflammasome and fibrosis-associated genes in the mWD-WD and mWD-CH-WD groups may lend support to this theory. Furthermore, the differences observed between mCH-WD versus mWD-WD and mCH-CH-WD versus mWD-CH-WD in these parameters suggests that in the context of MAFLD pathology, the mWD exposure may predispose the mice to more rapidly developing disease when they are challenged with WD compared with their counterparts lacking an mWD exposure. It should be noted that these mWD-induced changes appear to be largely related to altered gene expression as evidenced by RNA sequencing data.

Recent data indicate that exposure to maternal obesity results in increased KC number and leads to worsened MAFLD in offspring (10). In agreement with these data, we find that exposure to mWD resulted in increased KC numbers in mWD-CH and mWD-CH-CH offspring; however, in contrast to what was reported in Huang et al. (10), we find that continual challenge with WD reduced KC numbers compared with CH-fed controls. While KC loss is a common feature of liver diseases, it is typically observed at more advanced stages of disease characterized by fibrosis and reduced liver function diseases (19–21, 40–44). Although we see increased pro-inflammatory and pro-fibrotic gene expression in mWD-WD- and mWD-CH-WD-fed mice, picrosirius red staining was not different between any groups. Why we observe reduced KC numbers despite the mild phenotype is unknown, although the diet composition and length of maternal exposure in these studies prior to mating (42% fat, 2 weeks) differed from what was previously published (60% fat, 8 weeks). This may suggest that diet composition and/or length of exposure, rather than liver phenotype, is one of the major factors regulating KC accumulation.

It was previously reported that KC ontogeny was not impacted by maternal obesity (10). While our studies largely support this idea, we did find small but significant differences in TdT labeling (i.e., monocyte input) when comparing mWD-WD mice with mWD-CH controls. While TdT labeling was not different when comparing KCs isolated from mCH-CH- and mWD-CH-fed mice at 12 weeks of age, we found that TdT labeling was increased in mWD-CH-CH-fed mice compared with mCH-CH-CH-fed mice at 16 weeks of age, despite the fact that both groups experienced the same experimental conditions, with the exception of the time point that was analyzed. This suggests either the impact of mWD exposure on KC ontogeny in these mice becomes more pronounced as they age, or mWD can promote a KC-die off that begins around 12 weeks of age. The fact we did not find increased monocyte recruitment into the KC niche in our mWD-CH-WD mice was somewhat surprising as it has been reported that the reduced KC numbers observed in mouse models of MAFLD are associated with increased monocyte recruitment to fill the niche (20). The differences observed between these studies and previous data in regard to KC ontogeny may be driven by model-specific nuances. For example, in these studies, dams were fed a WD for only 2 weeks prior to mating, representing a model of maternal overnutrition, whereas dams were fed a high-fat diet (HFD) for 8 weeks prior to mating in published studies, representing a model of maternal obesity (10). Although it is tempting to speculate that a mWD/mHFD for longer periods would have a greater impact on KC ontogeny (due to maternal metabolic changes), there may also be compensatory effects that augment the offspring phenotypes when female mice are exposed to WD/HFD for prolonged periods prior to becoming pregnant. Future studies investigating the effects of specific diet components and the length of mWD/mHFD exposure are required to fully understand the impact that they have on KC numbers, ontogeny, and function.

In sum, we find that continuous challenge with WD following mWD exposure leads to enhanced expression of inflammatory and fibrosis-related genes, which is correlated with a greater reduction in KC proportion and TdT labeling than mCH offspring challenged with WD in adulthood (in relation to their respective maternal diet controls). Furthermore, we find that mWD alone results in increased KC number and slightly alters ontogeny, suggesting that perinatal exposure to WD alters KC development. We show that KCs isolated from mWD-CH-WD mice have increased expression of inflammatory genes, suggesting that perinatal exposure to WD imprints long-lasting alterations on KC development and response to subsequent challenges. Notably, the emergence of increased expression of the inflammasome gene *Nlrp3* and pro-fibrotic genes *Col1a1* and *Tgfb1* in young adult mice after just 4 weeks of post-weaning WD exposure (mWD-CH-WD) is surprising when considering adult WD fed models take up to 32 weeks to develop fibrosis (46). As expression of *Nlrp3* has been found to be significantly increased in MAFLD patients (39), these results suggest mWD exposure with WD challenge in adulthood may help promote MAFLD development in offspring. These studies support the idea that mWD exposure results in a lasting impact and demonstrate that changes in the perinatal period diet can have profound consequences on susceptibility to MAFLD (47), particularly when offspring are exposed to WD in adulthood.

## Data Availability

Bulk RNA sequencing data can be found in Gene Expression Omnibus repository under accession number GSE312316.All other original data generated from the study will be made available upon reasonable request to the corresponding authors.

## Supplementary material

Supplementary Figure 1 Diet compositions. **(A)** Table showing percent kilocalorie composition of each major nutrient group in the respective CH (PicoLab) and WD (Envigo) diets **(B)** Table detailing ingredients in each major nutrient group in the CH and WD fed during these studies.

Supplementary Figure 2 Maternal WD and post-natal WD exposure does not affect plasma insulin or glucose levels. **(A)** Weight (g) of offspring at time of harvest. **(B, C)** Plasma insulin **(B)** and glucose **(C)** levels at time of harvest. Data are shown as the means ± S.E.M., two-way ANOVA with Fishers LSD test. n=5–8 per group **(A)**, n=3–5 per group **(B**, **C)**. All male mice; each dot represents one animal.

Supplementary Figure 3 Gating strategy used to identify cell populations by flow cytometry.

Supplementary Figure 4 Maternal WD and post-natal WD re-challenge increases serum glucose in offspring. **(A)** Weight (g) of offspring at time of harvest across groups. **(B, C)** Plasma insulin **(B)** and glucose **(C)** levels at time of harvest. Data are shown as the means ± S.E.M., two-way ANOVA with Fishers LSD test. n=5–8 per group **(A)**, n=3–5 per group **(B**, **C)**. All male mice; each dot represents one animal.

Supplementary Table 1 Antibodies used in flow cytometry.

Supplementary Table 2 Primer sequences used for qPCR analysis.
