# Supplementary figures and images for: Maternal western-style diet alters Kupffer cell proportion leading to metabolic dysfunction-associated steatotic liver disease when challenged with western diet in adulthood
Source: Front Immunol. 2025 Dec 10;16:1698609. doi: 10.3389/fimmu.2025.1698609 (PMC12727619; doi:10.3389/fimmu.2025.1698609)

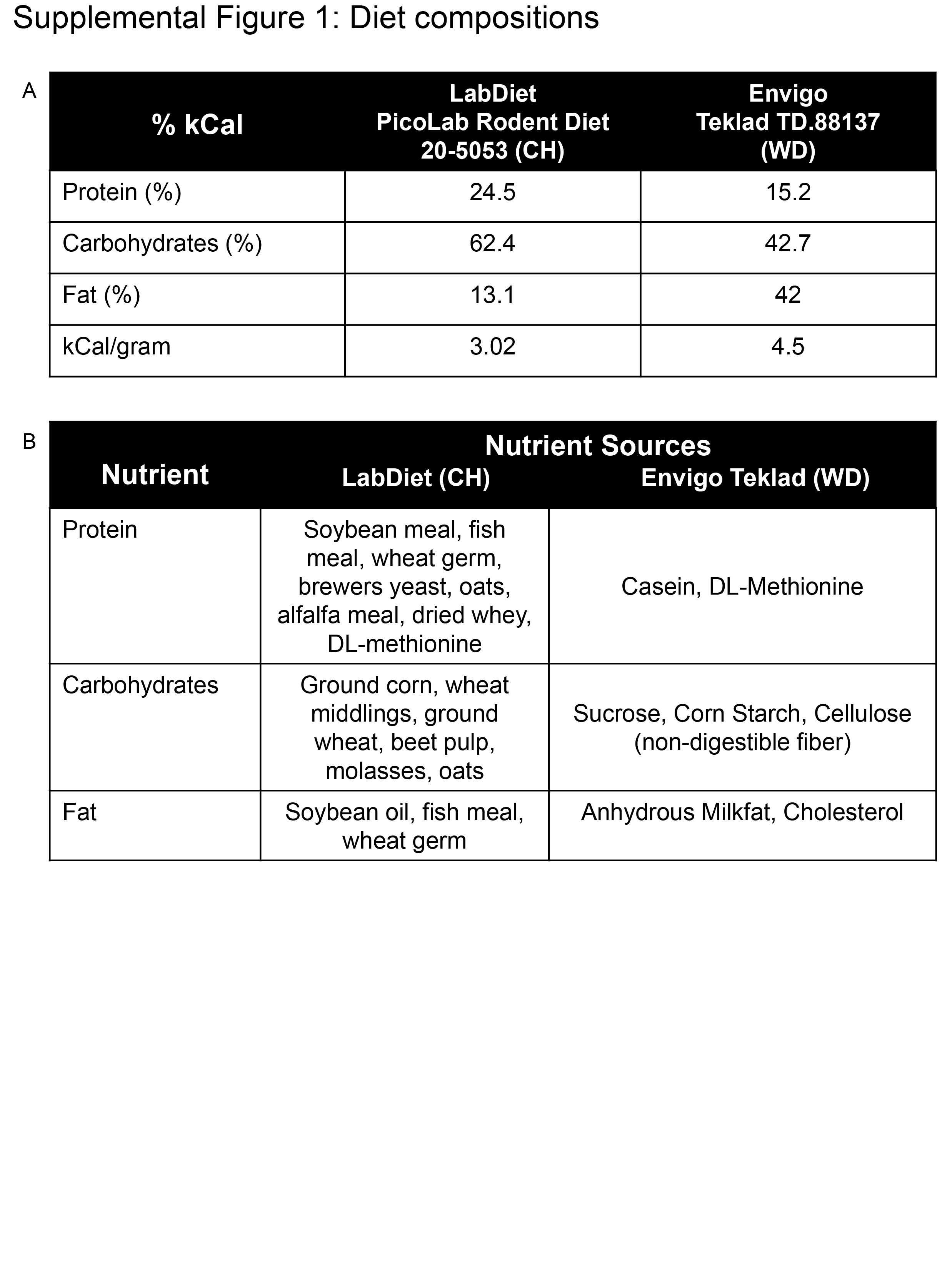

Supplement: Supplementary Figure 1 — Diet compositions. (A) Table showing percent kilocalorie composition of each major nutrient group in the respective CH (PicoLab) and WD (Envigo) diets (B) Table detailing ingredients in each major nutrient group in the CH and WD fed during these studies. [file Image1.jpeg]

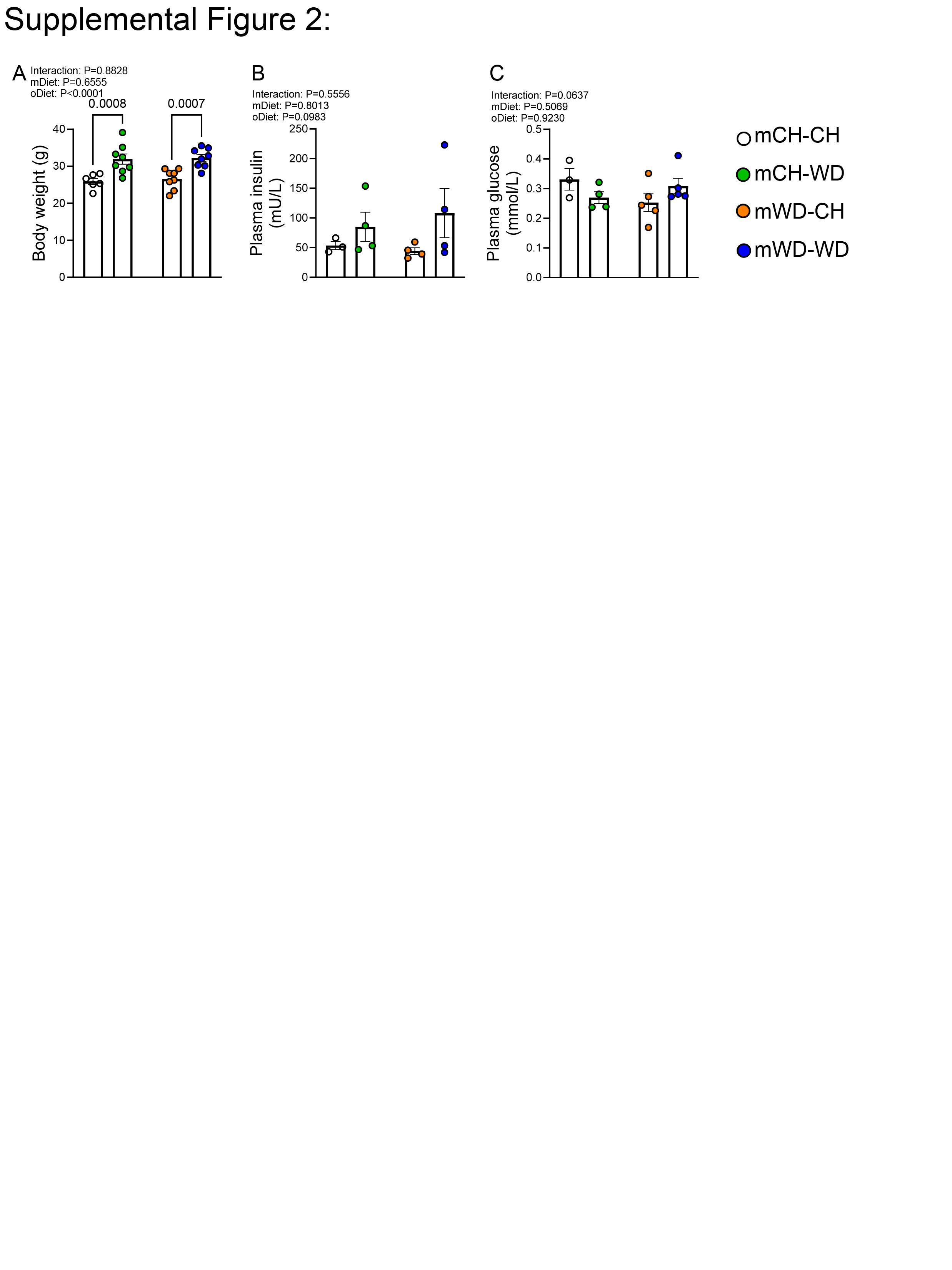

Supplement: Supplementary Figure 2 — Maternal WD and post-natal WD exposure does not affect plasma insulin or glucose levels. (A) Weight (g) of offspring at time of harvest. (B, C) Plasma insulin (B) and glucose (C) levels at time of harvest. Data are shown as the means ± S.E.M., two-way ANOVA with Fishers LSD test. n=5–8 per group (A), n=3–5 per group (B, C). All male mice; each dot represents one animal. [file Image2.jpeg]

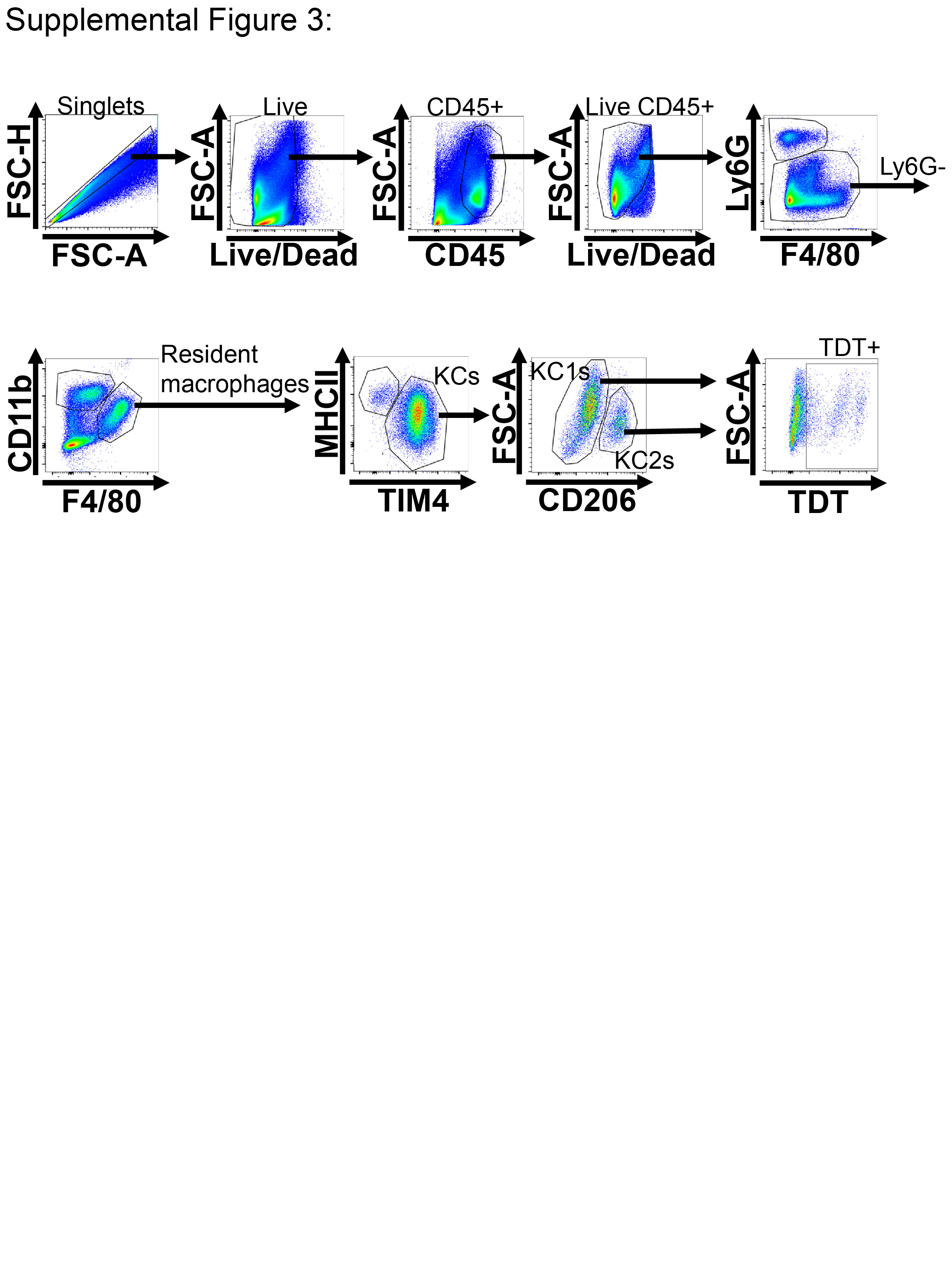

Supplement: Supplementary Figure 3 — Gating strategy used to identify cell populations by flow cytometry. [file Image3.jpeg]

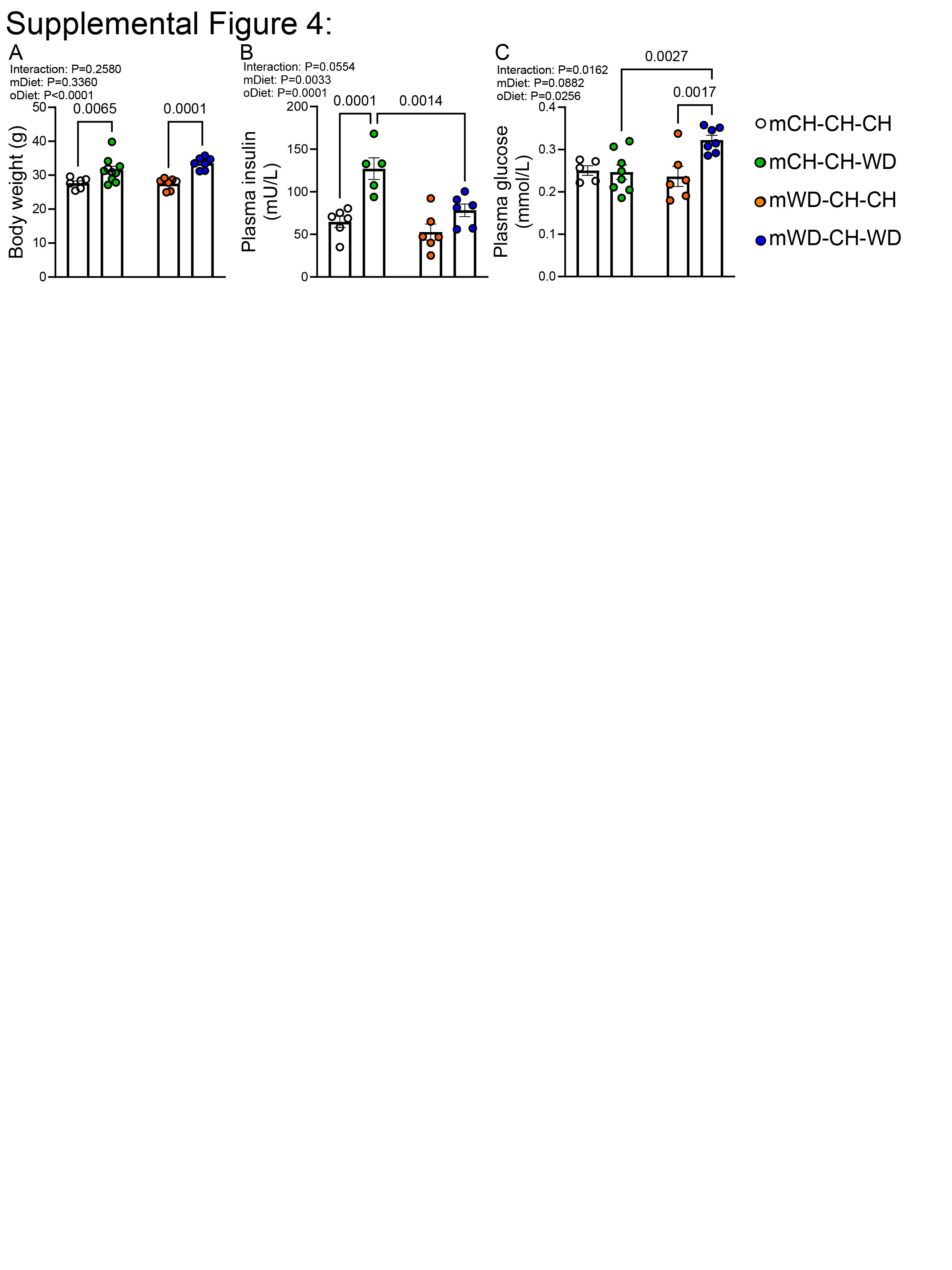

Supplement: Supplementary Figure 4 — Maternal WD and post-natal WD re-challenge increases serum glucose in offspring. (A) Weight (g) of offspring at time of harvest across groups. (B, C) Plasma insulin (B) and glucose (C) levels at time of harvest. Data are shown as the means ± S.E.M., two-way ANOVA with Fishers LSD test. n=5–8 per group (A), n=3–5 per group (B, C). All male mice; each dot represents one animal. [file Image4.jpeg]

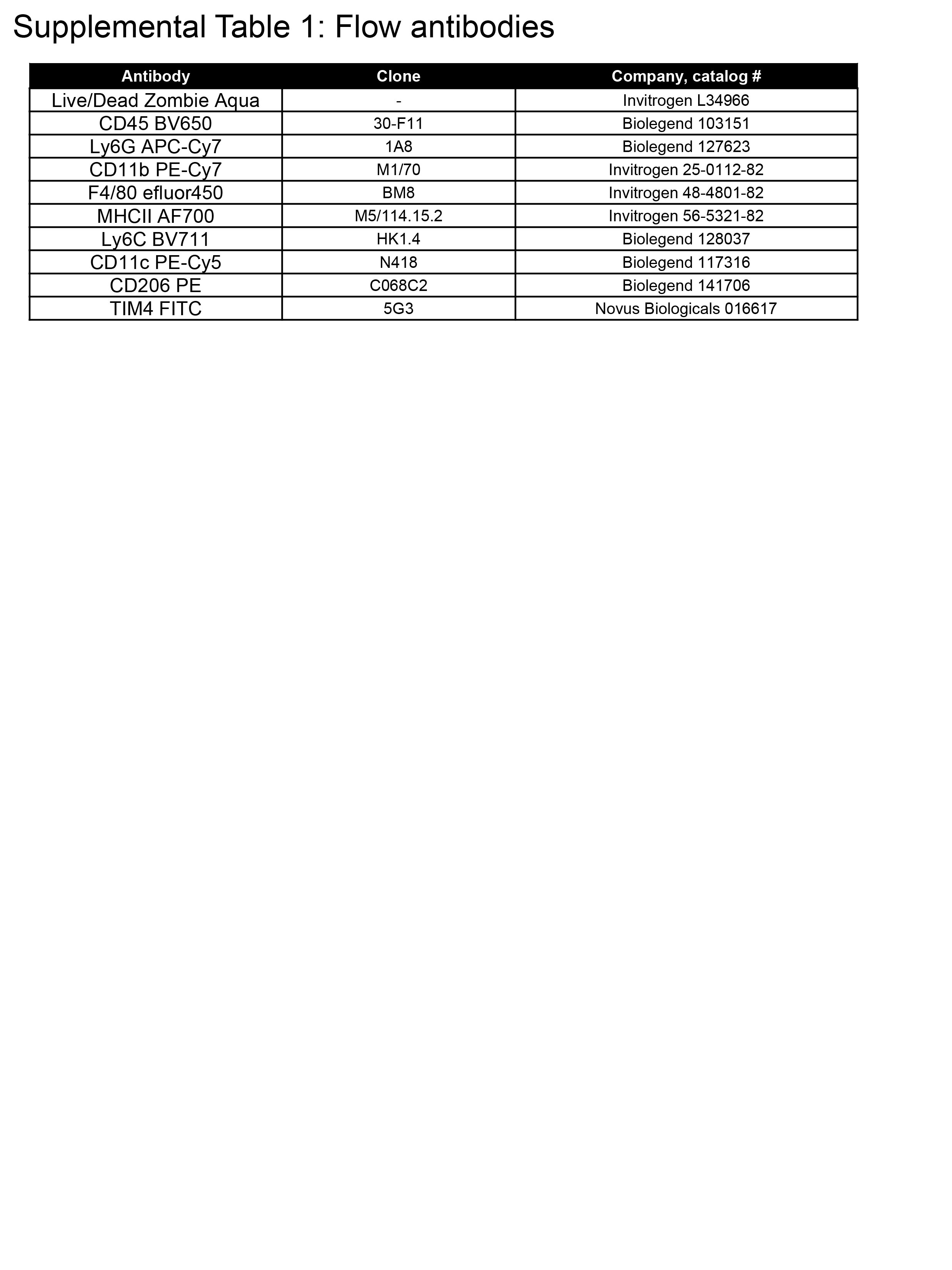

Supplement: Supplementary Table 1 — Antibodies used in flow cytometry. [file Image5.jpeg]

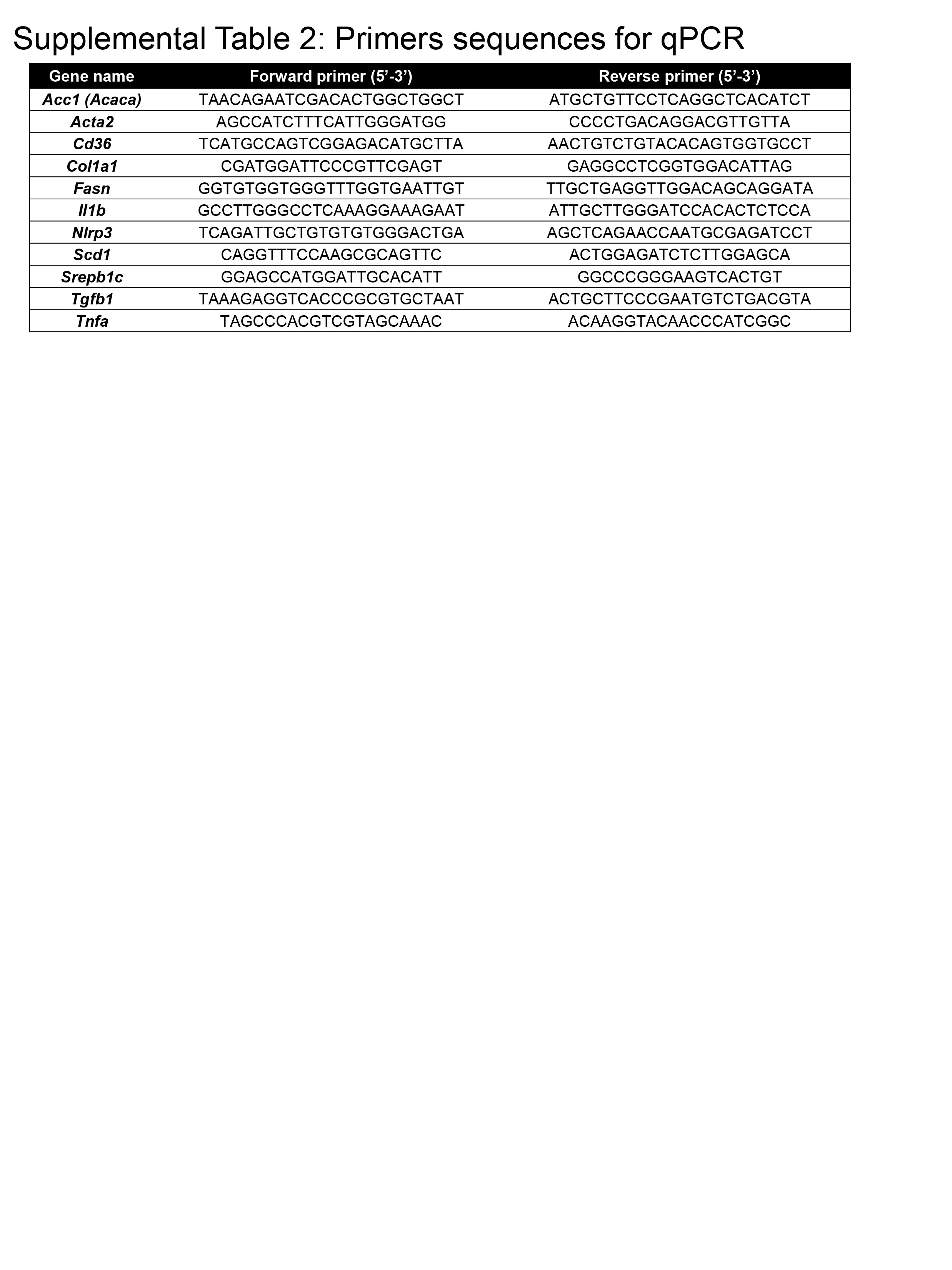

Supplement: Supplementary Table 2 — Primer sequences used for qPCR analysis. [file Image6.jpeg]
